# Versatile DIY Route for Incorporation of a Wide Range of Electrode Materials into Rotating Ring Disk Electrodes

**DOI:** 10.1021/acs.analchem.2c01744

**Published:** 2022-06-29

**Authors:** Joshua J. Tully, Zhaoyan Zhang, Irina M. Terrero Rodríguez, Lee Butcher, Julie V. Macpherson

**Affiliations:** Department of Chemistry, University of Warwick, Coventry CV4 7AL, U.K.

## Abstract

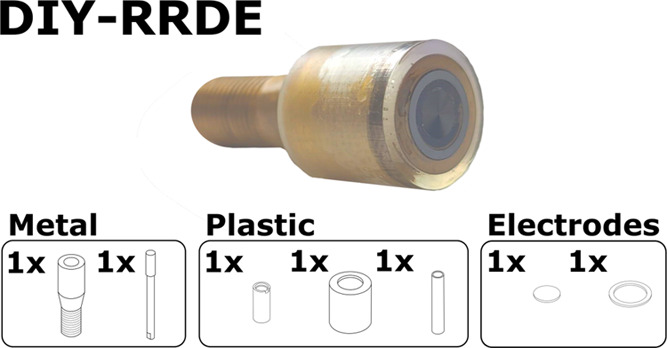

Rotating ring disk electrodes (RRDEs) are a powerful and versatile tool for mechanistically investigating electrochemical reactions at electrode surfaces, particularly in the area of electroanalysis and catalysis. Despite their importance, only limited electrode materials (typically glassy carbon, platinum, and gold) and combinations thereof are available commercially. In this work, we present a method employing three-dimensional (3D) printing in conjunction with machined brass components to produce housing, which can accommodate any electrode material in, *e.g*., pressed powdered pellet, wafer, rod, foil, or vapor deposited onto a conductive substrate form. In this way, the range and usability of RRDEs is extended. This custom do-it-yourself (DIY) approach to fabricating RRDEs also enables RRDEs to be produced at a significant fraction of the cost of commercial RRDEs. To illustrate the versatility of our approach, coplanar boron-doped diamond (BDD) RRDEs are fabricated for the first time using the approach described. Experimental collection efficiencies for the redox couple FcTMA^+^/FcTMA^2+^ are found to be very close to those predicted theoretically. BDD electrodes serve as an ideal electrocatalyst support due to their low background currents, wide solvent potential window in aqueous solution, and chemical and electrochemical stability in acid and alkali solutions. The BDD RRDE configuration is employed to investigate the importance of surface-incorporated nondiamond carbon in BDD on hydrogen peroxide generation *via* the oxygen reduction reaction in acid solutions.

## Introduction

The rotating ring disk electrode (RRDE) is a powerful electrochemical tool for the mechanistic analysis of electrochemical reactions at electrode surfaces.^1,2^ This is due to the ability of the RRDE to detect the products of an electron transfer reaction occurring at the inner disk electrode *via* the outer ring electrode.^2^ In this way, RRDEs can be employed to identify different mechanistic pathways in an electron transfer process.^3^ RRDEs, in particular, have found use as a tool for assessing electrocatalyst activity for processes such as the oxygen reduction reaction (ORR),^4−6^ nitrogen reduction reaction,^7^ and carbon dioxide reduction.^8^ The RRDE ring electrode can also be used as a pH sensor to detect pH changes resulting from reactions on the disk.^9,10^

In a RRDE, the ring and disk electrodes can be made from the same, or different, electrode materials, depending on the electron transfer process to be studied. For electrocatalytic studies, the electrode is the electrocatalyst or the electrocatalyst is deposited on the RRDE electrode (in *e.g.*, nanoparticle (NP) or ink form). For the latter, the prerequisite is that the electrode has significantly lower electrocatalytic activity than the deposited material and is noncorroding in the solution/potential region of interest and thus will not interfere with the reaction under study.^11,12^ The entire electrode system is typically encased in an insulating housing containing the contacts and threads required to attach and electrically connect the two electrodes to the rotating apparatus. As the RRDE is rotated, the solution is moved from the bulk toward the face of the disk electrode, which then exits parallel to the RRDE surface, flowing over the ring electrode as it moves outward toward the RRDE edge. The well-defined hydrodynamic flow not only enhances transport of electrogenerated species from the disk to the ring (compared to stationary conditions) but also enables the proportion of electroactive species collected at the ring, relative to that produced at the disk, to be predicted. This is defined as the collection efficiency (CE) and is an intrinsic property of each RRDE design with a dependence on the relative radii of the ring and disk electrodes.^2^

While commercial RRDEs are widely available, the electrode selection is typically limited to combinations of more common electrode materials such as gold, platinum, and glassy carbon (GC). Even with these standard materials, RRDEs can be costly (>$1.5k), and to extend the range of applications, there is a need to move to different electrode materials. For example, for electrocatalytic measurements, GC is often used as the disk electrode onto which the electrocatalyst is deposited, due to its reduced electrocatalytic activity compared to Pt and Au. However, GC is prone to dissolution at high oxidizing potentials, especially in alkaline solutions.^13^ Our approach expands the range of materials that can be used as both the disk and ring electrodes (for efficient detection of generated species). To the best of our knowledge, there are only two published papers on in-house-built RRDEs;^14,15^ however, the focus of both of these papers is on building an RRDE with a removable central disk. Furthermore, both papers provide only limited guidance on the design and construction processes. In contrast, this work aims to serve as an easy-to-follow guide for producing simple, yet effective RRDEs which are capable of housing a wide variety of electrode materials.

To prove the effectiveness of our approach to producing do-it-yourself (DIY) RRDEs, we focus on the production of coplanar boron-doped diamond (BDD) RRDEs, where the BDD is produced in a freestanding form, *via* chemical vapor deposition. To the best of our knowledge, no entirely BDD RRDEs have been made before (*i.e*., with BDD as both disk and ring electrode) most likely due to perceived difficulty. Thus, the work presented also exemplifies the versatility of the approach outlined. The method presented is applicable to any electrode material, which can be produced in pressed powdered pellet, wafer, rod, or foil form or which can be vapor (chemical/physical) deposited or sputtered/evaporated onto a conductive substrate. BDD has many useful electrochemical attributes in aqueous solutions, which include extremely low background currents, wide solvent window, high resistance to corrosion under both acid and alkali conditions,^16,17^ and an even lower electrocatalytic activity than other carbon electrodes, including GC.^16^ All of these properties make BDD an extremely useful electrocatalyst support electrode.^18^ The high oxygen evolution overpotential on BDD also allows for the more efficient anodic conversion of specific species to products, compared to other electrode materials.^19^

For this work, the DIY RRDE electrode was used to investigate the impact of nondiamond carbon (NDC) presence in BDD on the prevalence of ORR *via* the two-electron pathway.^20^ While NDC can occur in BDD as a result of the growth conditions employed, here we introduce NDC to the BDD surface, in a controllable way, using a nanosecond (ns) laser ablation process.^21−23^ This procedure has been used previously as a means of conferring increased electrocatalytic activity on a BDD electrode.^21,22^ While in previous studies a microdot NDC pattern in BDD was adopted,^21,22^ in this work, the entire surface was converted to NDC in order to exclusively investigate the role of NDC. To investigate the ORR mechanism (*i.e*., two-electron *vs* four-electron), the ring electrode was held at a sufficient oxidizing potential to detect hydrogen peroxide (H_2_O_2_), which is a product of the two-electron transfer pathway, eq 1(24,25)
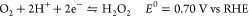
1To improve the electrocatalytic efficiency of the BDD ring electrode toward H_2_O_2_ oxidation, the surface of the ring was modified by electrochemical deposition of platinum NPs.^11^ Pt NPs on carbon materials (such as GC) have been used previously for the electrochemical detection of H_2_O_2_.^26,27^

## Experimental Section

### Electrode Preparation

BDD ring and disk electrodes were made from 360-μm-thick freestanding electroanalysis-grade BDD,^28^ supplied by Element 6 Ltd. (Oxford, U.K.). The growth face (used as the electrode face) was polished to *ca*. nanometer roughness and the nucleation face lapped to *ca*. micrometer roughness. The electrodes were cut from the wafer using a 355 nm Nd:YAG laser micromachining system (E-355-ATHI-O system, Oxford Lasers Ltd., U.K.) with a nominal pulse length of 34 ns. Cutting was performed in three passes using a trepan system with a fluence of 760 J cm^–1^. The disk electrode was either used as is or the surface laser-ablated with a fluence of 14 J cm^–1^ (*i.e*., just above the ablation threshold of BDD)^21^ in a spiral pattern to leave a layer of NDC covering the entire BDD surface.

Once the electrodes were cut, they were acid cleaned in concentrated H_2_SO_4_ (>96%, Fisher Scientific, U.K.) saturated with KNO_3_ (99%, Sigma-Aldrich, U.K.) and heated at 200 °C for 30 min. This was followed by another 30 min in 200 °C H_2_SO_4_ and then finally a wash in ultrapure water.^28^ This acid cleaning step removes any loose amorphous carbon (soot) from laser cutting, as well as aiding formation of a robust layer of NDC integrated into the BDD surface.^23^ For the BDD disk and ring electrodes, a subsequent thermal anneal at 600 °C for 5 h in air was used to reduce the amount of NDC remaining on the laser-cut surfaces (*i.e*., the sidewalls).^23^ A titanium (Ti:10 nm) and gold (Au:400 nm) contact was then sputtered (Moorfield MiniLab 060 Platform Sputter system) onto the lapped (nucleation) face of each electrode and annealed in air (400 °C for 5 h) to create an Ohmic contact.^29^

### 3D Printing

Nonconductive parts of the electrode body were designed in Fusion 360 (Autodesk) and printed on a Form 3 (FormLabs) stereolithography (SLA) three-dimensional (3D) printer using a standard clear resin (Formlabs) at a 50 μm layer height. Once printed, parts were washed for 10 min in isopropyl alcohol (IPA, Analytical Reagent Grade, ThermoFisher Scientific) to remove excess resin (Form Wash, FormLabs). This was followed by an additional 405 nm UV cure for 20 min at 60 °C to achieve the full material properties of the resin (Form Cure, FormLabs).

### Electrochemical Characterization

A 760E CHI BiPot (CH Instruments, Texas) was used for electrochemical measurements alongside a saturated calomel reference electrode (SCE, CH Instruments Inc.) and Pt coil counter electrode. All solutions were prepared in ultrapure water (>18.2 MΩ cm, Milli-Q, Millipore Corp.) and measurements made under ambient conditions. Aqueous solvent potential window measurements were performed by cyclic voltammetry (CV) in 0.1 M potassium nitrate (KNO_3_, 99%, Sigma-Aldrich, U.K.). Redox mediator measurements were performed by CV in a solution of 1 mM (ferrocenylmethyl)trimethylammonium (FcTMA^+^) in 0.1 M KNO_3_. Uncompensated resistance (*R*_u_) measurements were conducted using chronoamperometry with 0.1 V steps, the nonfaradic decay was then fitted to extract *R*_u_.^30,31^ Determination of the collection efficiency was performed by running linear sweep voltammetry (LSV) measurements on the disk electrode and recording the current–time (*i*–*t*) response on the ring electrode.

### Pt Deposition

The protocol for Pt NP deposition on the BDD ring electrode was adapted from Hutton et al.^11^ The electrode was held at −1.00 V *vs* SCE in a solution of hexachloroplatinate (K_2_PtCl_6_, 99%, Sigma-Aldrich, U.K.) in 0.1 M hydrochloric acid (HCl, 99%, Sigma-Aldrich, U.K.) for 300 s. During deposition, the RRDE was rotated at 1500 rpm to minimize the hydrogen gas produced on the ring electrode blocking the electrode surface.

### Hydrogen Peroxide Experiments

After Pt deposition, the ring electrode response to H_2_O_2_ (30% w/w, Sigma-Aldrich, U.K.) in 0.1 M perchloric acid (HClO_4_, 70%, Sigma-Aldrich, U.K.) was calibrated. The RRDE electrode was rotated at 2000 rpm, the ring held at 1.00 V *vs* SCE, and the current response recorded for 30 s at each H_2_O_2_ concentration. This potential was chosen to give a sufficiently large overpotential for H_2_O_2_ oxidation, but before the oxidation of water starts to compete (*vide infra*).^26,27^ The Pt NP-BDD ring was calibrated for 11 concentrations in equal increments of 0.14 mM in the range 0–1.4 mM. The upper end was chosen by considering the saturation point of O_2_ in water to be 1.22 mM.^32^ Approximately, this value was assumed to be the dissolved O_2_ concentration in the O_2_-saturated HClO_4_ solution. Assuming a 1:1 ratio and 100% conversion (eq 1) means the maximum concentration of H_2_O_2_ that can be produced is ∼1.22 mM. The current average over the last 5 s of the chronoamperometric response for each concentration was used to construct the current *vs* H_2_O_2_ concentration calibration plot.

After calibration, the RRDE was used to perform generation-collection experiments in 0.1 M HClO_4_ at 2000 rpm. The disk electrode was scanned from 0.00 to −0.80 V *vs* SCE, a region where ORR should be observed, at a scan rate of 5 mV s^–1^, with the Pt NP-BDD ring electrode held at 1.00 V *vs* SCE. After a generation-collection experiment, the H_2_O_2_ calibration was repeated. To create an O_2_-saturated solution, O_2_ (99.5% purity, BOC, U.K.) was bubbled through a solution of 0.1 M HClO_4_ for a period of 60 min (assuming 2 min for every milliliter of solution). During the experiment, O_2_ was flowed across the top of the solution to maintain saturation.

## Results and Discussion

### Fabrication

**Figure 1:**
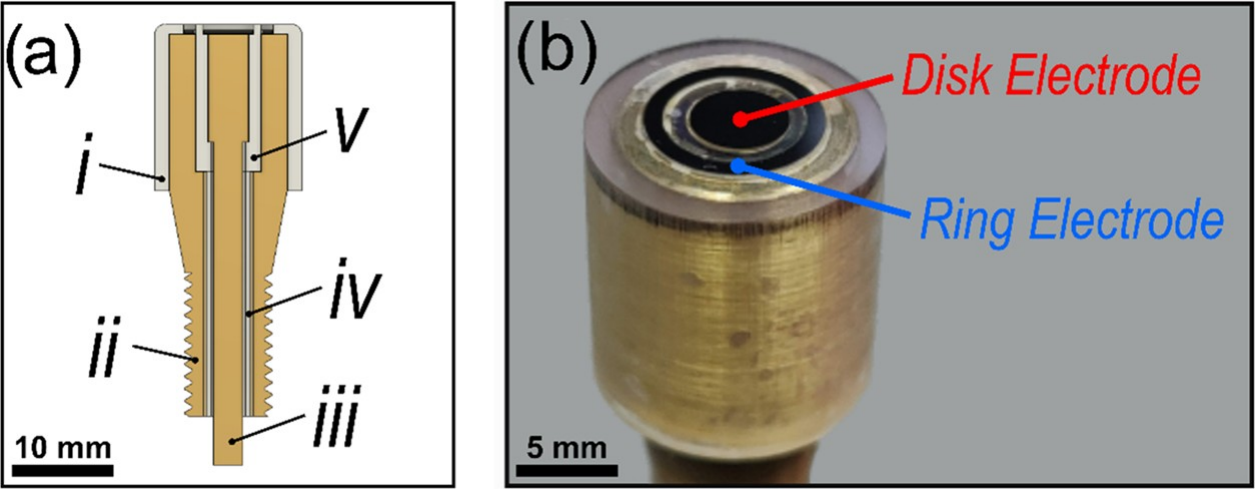
(a) Rendering of a cross section through the center of the RRDE electrode, showing the arrangement of the ring and disk contacts. The five components are labeled as (i) insulating outer case; (ii) brass outer core; (iii) brass inner core; (iv) insulating tube; and (v) insulating spacer. (b) Photograph of an assembled RRDE containing BDD ring and disk electrodes.

An RRDE with a 5.00 mm diameter disk and a ring with inner and outer diameters of 7.00 and 9.00 mm, respectively, was chosen for this work. These electrode sizes are common in commercial RRDEs. A slightly larger ring disk gap (1.00 mm as compared to 0.50 mm) has been adopted to make the DIY fabrication process easier, although smaller gaps are also possible. In this work, the RRDE is designed to integrate with a commercial rotator, here a Pine Research Modulated Speed Rotator; however, the designs could be easily modified for any rotator system. The RRDE itself consists of five components (Figure 1a), which can be used with electrode materials in formats such as rod, pressed powdered pellet, wafer, foil, *etc*. The five components include: (i) the insulating outer case, the top surface of which lies coplanar with the ring and the disk; (ii) the brass outer core, which serves as both the ring contact and main body; (iii) the brass inner core, which is used as the disk contact; (iv) the insulating tube, which separates and spaces the brass inner and outer cores; and (v) the insulating spacer, which prevents the two brass parts from touching. While the brass parts are made using traditional machining techniques on a lathe, the insulating parts are designed to be printable using SLA 3D printing.^33^

Once all parts have been fabricated, the RRDE can be assembled using basic tools and equipment. First, the five components (i–v) are placed in their appropriate positions and held together with rapid epoxy (Araldite Rapid). The electrodes are then integrated into this assembled housing by locating the ring and disk electrodes in their respective recesses and holding them in place using a conductive epoxy (Conductive Epoxy, Chemtronics), which forms an electrical contact to the brass. Once the conductive epoxy is set, the entire RRDE surface is then flooded with a low-viscosity insulating epoxy (Standard Clear, FormLabs). A low-viscosity epoxy is preferable as it flows more easily into gaps between the electrode and housing, preventing voids. Any remaining epoxy on the electrode surfaces is polished off using abrasive papers (CarbiMet, Buhler). Care should be taken to choose a paper that is softer than the electrode material being used, to prevent damage to the electrode. A more detailed assembly guide, including possible approaches for dealing with electrodes when supplied in different forms, editable computer-aided design (CAD) files, workshop drawings, and files suitable for 3D printing, can be found in SI1. Note, if processes involving gas evolution were to be studied, it may be advantageous to make the insulating outer case and spacer from a hydrophilic material to reduce bubble adhesion.^34^ However, it is important to ensure that the epoxy resin used for sealing adheres well to the insulating material utilized.

The approach described results in a cost-effective RRDE, where the five components (i–v) can be fabricated and assembled for as little as $25. SI2 shows the typical cost of component parts. A completed coplanar BDD RRDE used in this work can be seen in Figure 1b. Two RRDEs were assembled for the studies described, which differed only in the disk electrode used: one employed a BDD disk, and the other a BDD disk where the surface had been converted to NDC *via* laser ablation.^35^ Both contained BDD ring electrodes.

### RRDE Characterization

To verify that the individual electrodes of RRDEs were functioning appropriately, the CV responses of the two BDD rings, one BDD disk, and one NDC–BDD disk, were recorded in 1 mM FcTMA^+^ in 0.1 M KNO_3_ at 100 mV s^–1^, under stationary conditions. Peak-to-peak separations, Δ*E*_p_, in the range of 69–88 mV were measured (SI.3). *R*_u_ measurements recorded in a solution of 0.1 M KNO_3_, using chronoamperometry,^31^ in a nonfaradic region of the CV (0.0 to 0.1 V *vs* SCE *n* = 5) were used to assess any possible contact resistance issues.^30,31^ All of the electrodes gave low *R*_u_ values <115 Ω (SI.4), suggesting that the electrodes were appropriately contacted. Due to the magnitude of the currents passed, Ohmic drop is not insignificant, thus both Ohmic drop and charge transfer resistance^36,37^ are likely to play a role in the observed Δ*E*_p_ values.

The surface roughness of the disk electrodes was characterized by white light interferometry (WLI; SI5) to understand the effect of laser ablation on the surface morphology and to provide a measurement of the electrode surface area. *Via* WLI, the BDD disk was shown to be smooth with an RMS of 10 nm. Incorporation of NDC into the surface by laser ablation increased the roughness of the disk to 460 nm RMS. The electrochemical response of quinones present in the laser-ablated surface was used to provide an indication of the NDC content of the surface (SI6).^35^ It has been previously shown that there is a direct relationship between the quinone surface coverage and the amount of NDC present in BDD electrode surfaces.^35^ Voltammetric measurements revealed that the NDC–BDD disk had two orders of magnitude more quinone groups in the surface than in the bare BDD disk. The presence of quinones in the BDD disk is likely to be due to a small contribution of NDC from the laser-cut disk edge.^36^

**Figure 2:**
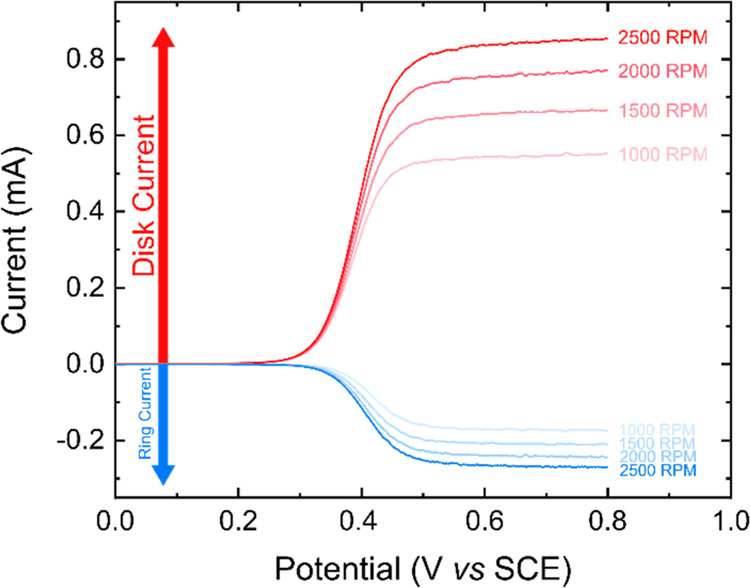
LSVs on a BDD disk electrode recorded at 100 mV s^–1^ in an aerated solution of 1 mM FcTMA^+^ in 0.1 M KNO_3_ (red lines), with the BDD ring electrode held at 0.05 V *vs* SCE (blue lines), for RRDE rotation rates of 1000, 1500, 2000, and 2500 rpm.

The collection efficiency of RRDEs for the redox couple FcTMA^+^/FcTMA^2+^ was measured for comparison with the theoretical efficiency for both the BDD (Figure 2) and NDC–BDD disk (SI7) RRDEs. In both the experiments, the potential of the disk electrode was swept from a value where no electron transfer occurred (0.00 V *vs* SCE) to one where the oxidation of FcTMA^+^ to FcTMA^2+^ was mass transport limited (red lines). The potential of the BDD ring was held at a value where reduction of FcTMA^2+^ was also mass transport limited (0.05 V *vs* SCE; blue lines). The experiments were performed as a function of rotation rate from 1000 to 2500 rpm in 500 rpm steps.

From these generation-collection experiments, the empirical collection efficiency (*N*_empirical_) of each RRDE was calculated by taking the ratio of the disk-to-ring currents in the limiting current region (0.60 V *vs* SCE), in accordance with eq 2.^2,38^ Values for the disk and ring currents can be found in SI8
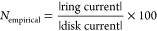
2Based on the disk (diameter = 5.00 mm) and ring dimensions (inner and outer diameters being 7.00 and 9.00 mm, respectively), the theoretical current efficiency^38^ was 35%. The BDD disk and ring RRDE has an empirical collection efficiency of 32%, close to the theoretical value. The NDC–BDD disk and BDD ring RRDE electrode has a very slightly lower empirical collection efficiency of 31%. The disk electrode currents for both RRDEs were also plotted against the rotation rate^1/2^, resulting in a good linearity (*R*^2^ = 0.999). Levich analysis^39^ enabled values of 5.1 × 10^–6^ and 4.9 × 10^–6^ cm^2^ s^–1^ (for BDD and NDC–BDD disk electrodes, respectively) to be extracted for the diffusion coefficient of FcTMA^+^. These values are in good agreement with the value measured (5.1 × 10^–6^ cm^2^ s^–1^) from the limiting current response recorded using a 25 μm diameter platinum ultramicroelectrode in the same solution (SI9).

### Case Study: ORR and Hydrogen Peroxide Generation

**Figure 3:**
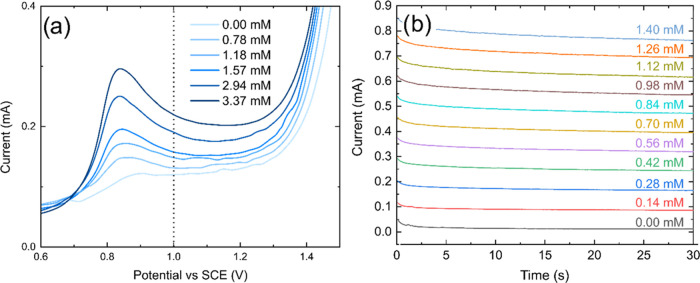
(a) Forward scan of CVs on the Pt NP-BDD ring in 0.1 M HClO_4_ with increasing concentrations of H_2_O_2_ from 0.00 to 3.37 mM. Dotted line: the potential chosen (1.00 V) for the ring in H_2_O_2_ generation-collection experiments. (b) Pre-ORR *i*–*t* calibrations for H_2_O_2_ recorded on the Pt NP-BDD ring of the RRDE, with a BDD disk electrode, rotated at 2000 rpm. The Pt NP-BDD ring electrode was held at 1.00 V *vs* SCE and H_2_O_2_ added *via* standard addition. The current over the last 5 s was averaged to give the calibration plot in SI11.

Initially, the detection of H_2_O_2_ was trialed on the BDD ring; however, no H_2_O_2_ peak was observed (data not shown). Thus, before carrying out ORR measurements, it was first necessary to verify that the BDD ring electrode could be sensitized toward H_2_O_2_ detection. Sensitization was achieved by electrodeposition of Pt NPs on the ring surface (see Experimental Section). After electrodeposition, SEM imaging revealed a high-density of Pt NPs tens of nanometers in size on the electrode surface (SI10). The response of the Pt NP-BDD ring electrode to H_2_O_2_ in 0.1 M HClO_4_ was first studied under stationary conditions to identify the H_2_O_2_ oxidation peak (Figure 3a) for a range of H_2_O_2_ concentrations ([H_2_O_2_]) from 0.00 to 3.37 mM. The CV commenced at 0.60 V *vs* SCE and was scanned positive to 1.50 V *vs* SCE and then negative to −0.20 V *vs* SCE. Three CVs were recorded per H_2_O_2_ concentration. To focus on the H_2_O_2_ oxidative response, the oxidative window only for each concentration is presented in Figure 3a (the third CV is presented).

The CVs revealed an oxidative peak at ∼0.85 V *vs* SCE, which increased with H_2_O_2_ concentration, demonstrating good correlation (*R*^2^ = 0.993, data not shown). A ring potential of 1.00 V *vs* SCE was chosen for H_2_O_2_ generation-collection experiments as a compromise between providing sufficient overpotential to drive the oxidative reaction but free from any currents associated with the electrooxidation of water on Pt NPs. The value of 1.00 V *vs* SCE is also similar to that used in the literature for H_2_O_2_ oxidation on Pt.^4,40,41^

[H_2_O_2_] calibration experiments were recorded immediately after electrodeposition of Pt NPs, as detailed in the Experimental Section. Exemplar *i*–*t* transients as a function of increasing [H_2_O_2_] are shown in Figure 3b. A very small decrease in current is observed with increasing time. For this reason, the current value taken to construct the current *vs* [H_2_O_2_] calibration plot was obtained by averaging the current over the final 5 s of the *i*–*t* transient. Generation-collection experiments to study ORR were then performed and the ring electrode calibrated again for [H_2_O_2_] post experiment. Each ORR generation-collection experiment was only considered valid if the pre- and postcalibration gradients (in mA mM^–1^) were within 10% of each other. In this way, it was possible to ensure that there had been no impactful deterioration in the ability of the Pt NP-BDD ring electrode to detect H_2_O_2_ quantitatively during the course of the ORR–H_2_O_2_ RRDE measurements. For RRDE experiments, the precalibration gradient was employed to calculate the concentration of H_2_O_2_ detected at the ring electrode. Note in order to convert this concentration to [H_2_O_2_] generated on the disk electrode it is necessary to multiple the former by (1/*N*_empirical_). Before and after [H_2_O_2_] calibrations of the Pt NP-BDD ring electrodes for both RRDEs can be found in SI11 (BDD disk) and SI12 (NDC–BDD disk), respectively.

**Figure 4:**
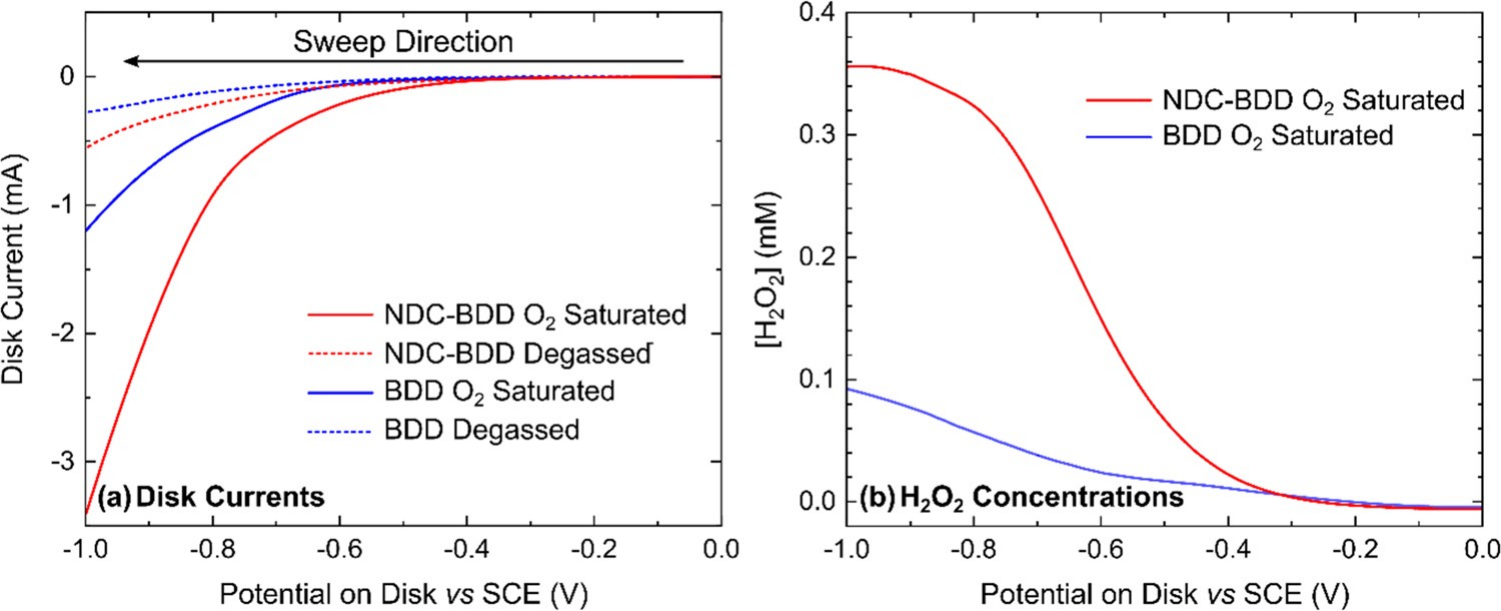
Generation-collection experiments conducted at 2000 rpm in 0.1 M HClO_4_ on BDD and NDC–BDD (disk) and Pt NP-BDD (ring) RRDEs in oxygen-saturated and deaerated conditions: (a) LSVs on the NDC–BDD and BDD disk electrodes, scanned at 5 mV s^–1^. (b) Generated [H_2_O_2_] on the disk electrode *vs* disk potential for the oxygen-saturated solution.

For ORR, the two disk electrodes were scanned over a reductive potential range from a value where no current flows (at 0.00 V *vs* SCE) into the start of the reductive solvent window (−1.00 V *vs* SCE) in both O_2_-saturated and deaerated 0.1 M HClO_4_ (Figure 4a). The Pt NP-BDD ring electrode was held at +1.00 V throughout to detect any H_2_O_2_ formed during ORR. The *y*-axis of Figure 4b represents the concentration of H_2_O_2_ generated on the disk electrode.

For the NDC–BDD disk, in the presence of saturated dissolved O_2_ (solid red line), when comparing against the deaerated solution response (dashed red line), ORR appears to commence just before the reduction of protons (0.1 M HClO_4_) starts to dominate the LSV response, the latter occurring *ca*. −0.75 V *vs* SCE. Thus, no steady-state response for ORR is observed. This is not surprising as ORR is known to be sluggish on carbon surfaces in acid electrolytes.^20^ However, ORR becomes much clearer when observing [H_2_O_2_] (presented as the concentration generated on the disk electrode) as a function of disk potential; solid red line in Figure 4b. Here, [H_2_O_2_] can be seen to increase from 0 mM, at a disk potential of 0.00 V *vs* SCE to a maximum, constant value of ∼ 0.36 mM, for disk potentials of *ca*. −1.00 V *vs* SCE. Thus, the simultaneously recorded [H_2_O_2_] generation data enables the potential at which ORR begins, on the disk electrode, to be more clearly identified. On the BDD disk electrode in the O_2_-saturated solution (blue solid line, Figure 4a), the LSV response toward ORR appears even more catalytically retarded. This is again in agreement with the [H_2_O_2_] data (blue line, Figure 4b), which shows a notable rise in concentration only when the potential is made more negative of *ca*. −0.60 V *vs* SCE. However, at −1.00 V *vs* SCE, [H_2_O_2_] has only reached a value of 0.9 mM. This data highlights the ability of NDC to increase the electrocatalytic activity of the BDD surface toward ORR and also shows the prominence of the two-electron transfer pathway.

Faradic efficiency (FE) for H_2_O_2_ generation at each electrode was calculated at potentials of −0.60, −0.70, and −0.80 V *vs* SCE according to eq S3 in SI.13. For both disk electrodes, the highest FE is seen at the least negative potential (−0.60 V *vs* SCE) with 26% for the BDD electrode and 64% for the NDC-modified BDD electrode. As the potential becomes increasingly negative, the FE decreases to 30% (for NDC) and 1% (for BDD) at −0.80 V. This is not surprising as proton reduction (0.1 M HClO_4_) is now significantly competing with ORR on both the electrodes. Determining mechanistic pathways for ORR is far simpler when the ORR process is isolated from the electrolyte reduction reaction. Higher FEs are seen for the NDC–BDD electrode as the disk potentials are made less negative; however, the ring currents are also much closer to the background currents in these regions.

## Conclusions

This paper describes a DIY guide incorporating 3D printing to constructing a RRDE using any electrode material that can be produced in rod, wafer, or foil form. It is also extendable to materials made in powder form that can be pressed into a pellet or materials that can be deposited onto a conductive substrate by processes such as physical/chemical vapor deposition and sputtering/evaporation. Electrodes fabricated by these methods can also be modified in conventional ways, such as drop casting or electrodepositing electrocatalysts onto the surface. This approach thus widens the range of possible electrode materials usable in the RRDE set-up, increases accessibility of the technique, and enables a larger range of RRDE applications, especially in the electrocatalysis field.

The DIY approach is illustrated *via* the first-time construction of a RRDE containing both coplanar BDD disk and ring electrodes. The collection efficiency of the BDD RRDE was shown to be very close to that theoretically predicted (using a simple redox species FcTMA^+^/FcTMA^2+^). The impact of the presence of NDC in BDD on its electrocatalytic activity toward ORR in an acid electrolyte was investigated by employing NDC–BDD disk electrodes in the RRDE set-up. Sensitization of the BDD ring electrode toward H_2_O_2_ detection was achieved by functionalizing with electrodeposited Pt NPs. Even though the ORR signal on the NDC–BDD disk was mostly obscured by that from proton reduction, a clear response for H_2_O_2_ detection was observed on the ring. The concentration of generated H_2_O_2_ on the ring electrode was shown to increase with increasing disk electrode potential, until a limiting value was reached. Over the same potential range, the bare BDD disk showed much more sluggish electroactivity toward ORR. The ORR signal is barely discernible, and reduced concentrations of H_2_O_2_ are detected. This data also highlights the use of BDD as an excellent electrocatalyst support material given its very low electrocatalytic activity. For work requiring acid or alkaline solutions and high oxidizing potentials, BDD also serves as an ideal corrosion-free support.

## Supporting Information Available

Design and step-by-step assembly instructions for RRDE electrodes (SI1); bill of materials (SI2); CVs of RRDE electrodes in 1 mM FcTMA^+^ and 0.1 M KNO_3_ (SI3); uncompensated resistance measurements (SI4); white light interferometry (WLI) of disk electrode surfaces (SI5); quinone surface coverage (QSC) measurements (SI6); generation-collection response of the NDC–BDD disk and BDD ring electrode (RRDE) (SI7); ring and disk currents at 0.60 V *vs* SCE (SI8); Levich analysis (SI9); SEM of Pt NP-modified ring electrode (SI10); BDD electrode ring calibrations for H_2_O_2_ (SI11); NDC–BDD electrode ring calibrations for H_2_O_2_ (SI12); and faradic efficiency (SI13) (PDF)

## Notes

All raw data used in preparation of this manuscript can be found at http://wrap.warwick.ac.uk/166752. These data are provided free of charge.
