# Supplementary material for: Versatile DIY Route for Incorporation of a Wide Range of Electrode Materials into Rotating Ring Disk Electrodes
Source: Anal Chem. 2022 Jun 29;94(27):9856–62. doi: 10.1021/acs.analchem.2c01744 (PMC9280712; doi:10.1021/acs.analchem.2c01744)
Supplement: Supplementary file 1 — ac2c01744_si_001.pdf [file ac2c01744_si_001.pdf]

Supporting Information

A Versatile DIY Route for Incorporation of a Wide Range of Electrode Materials into Rotating Ring Disk Electrodes

Joshua J. Tully[1*], Zhaoyan Zhang[1], Irina M. Terrero Rodríguez[1], Lee Butcher[1], and Julie V. Macpherson[1*]

[1]Department of Chemistry, University of Warwick, Coventry, CV4 7AL, UK

Contents:

ESI.1: Design and Step-By-Step Assembly Instructions for RRDE Electrodes

ESI.2: Bill of Materials

ESI.3: CVs of RRDE Electrodes in 1 mM $FcTMA^+$ and 0.1 M KNO_3

ESI.4: Uncompensated Resistance Measurements

ESI.5: White Light Interferometry (WLI) of Disk Electrode Surfaces

ESI.6: Quinone Surface Coverage (QSC) Measurements

ESI.7: Generation Collection Response of the NDC-BDD disk and BDD ring electrode (RRDE)

ESI.8: Ring and Disk Currents at 0.60 V *vs* SCE

ESI.9: Levich Analysis

ESI.10: SEM of Pt-NP Modified Ring Electrode

ESI.11: BDD Electrode Ring Calibrations for H_2O_2

ESI.12: NDC BDD Electrode Ring Calibrations for H_2O_2

ESI.13: Faradic Efficiency

Supporting Information

ESI.1 Design and Step-By-Step Assembly Instructions for RRDE Electrodes

The RRDE electrodes used in this work were fabricated in house using the ten-step process outlined below and in Figures S1.3- S1.6. Files and drawings for the parts used in this paper are available on request from the corresponding authors.

Design and Fabrication:

The housing of the RRDEs consist of five parts, which can then be assembled as needed without the need for specialist tools or equipment. Three of these parts have been designed such that they can be printed on a SLA 3D printer to lower costs, if needed. The remaining two brass parts are designed to be fabricated on a lathe as most research institutions have access to mechanical workshops. If no access to a mechanical workshop is possible the manufacture of these parts could be outsourced, or a metal selective laser sintering 3D printer could be used. The recesses in the insulating parts should be sized appropriately for the thickness of the electrode material employed. Generally, it is ideal to have ca. 300 µm of additional depth to make sure that there is enough epoxy resin to seal the electrodes, but as little as an additional 100 µm can be used. The design used in this article can accommodate an electrode 360 µm thick, and a recess 600 µm deep, is employed. If needed, a sixth component can also be produced which acts as a stand to ensure the ring and disk electrodes remain planar during construction.

Thread Sizing:

One of the critical components to get right when integrating a custom RRDE electrode into a commercial rotator system is to ensure that the threads match so that everything can screw together. The method of thread sizing is slightly different for metric (commonly used in Europe) or imperial (commonly used in the USA) so you first have to decide what type of thread you are likely to have based on the supplier of your rotator. In both cases it is easier to

size a male thread, but it can also be done with a female. If the thread dimensions are not known, follow the below procedure.

Metric: Place the thread up against a ruler and measure the distance from one thread to the next, this is the thread pitch *i.e.* how far up or down the thread moves with each full rotation. Next, measure the diameter of the thread and put an M in front of it. From here you can create a thread classification that should be of the format diameter followed by thread pitch (*i.e.* M4 × 0.5). If this thread corresponds to a standard combination the easiest way to check is to screw the opposite gender of thread into it. A mechanical workshop will likely have pitch gauges which will allow them to do this more accurately, although this method works well in the absence of more specialized equipment. A schematic representation of this can be found in Figure S1.1.

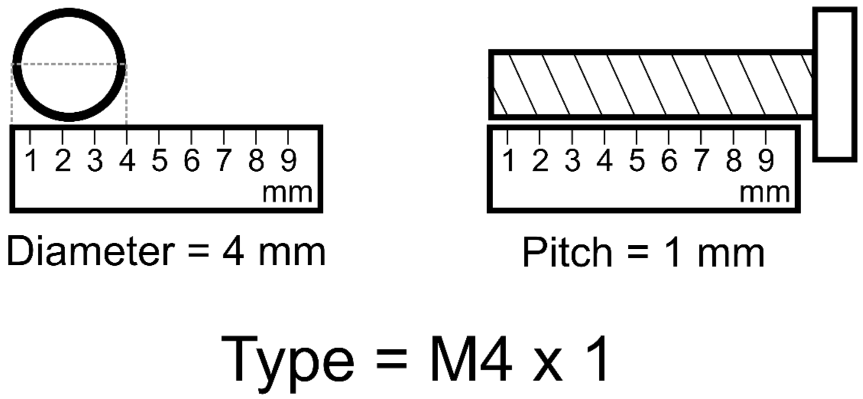

Figure S1.1: Schematic showing how to size metric threads without a pitch gauge.

Imperial: Measure the diameter in inches. Then, count the number of threads per 1 inch in length of the threaded part, imperial threads are defined as threads per inch (TPI). Write out the thread classification which should be the diameter followed by the TPI (i.e. $3/16^{th}$ × 32). Again, if this thread corresponds to a standard combination the easiest way to check is to screw the opposite gender of thread into it. A schematic representation of this can be found in Figure S1.2.

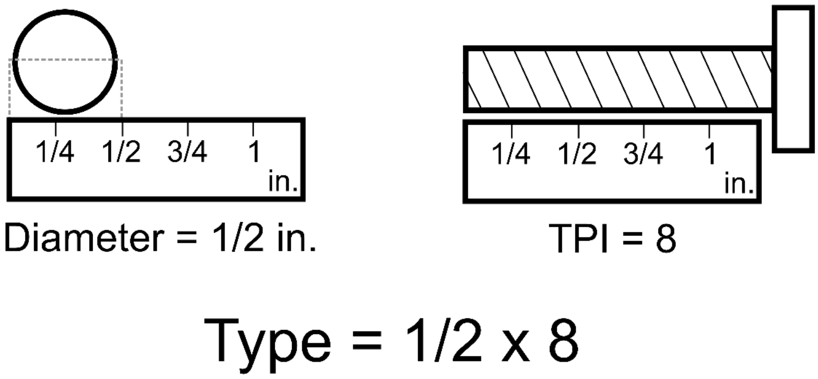

Figure S1.2: Schematic showing how to size imperial threads without a pitch gauge.

Assembly:

This section describes the assembly process for a RRDE electrode consisting of two BDD electrodes which are 360 µm thick and polished on the top surface to ~ nm roughness. Advice on how to modify the assembly method for other materials is detailed later in this section.

1. Collect all the prepared parts for the RRDE including (i) the insulating outer case (which prevents the brass being exposed to the solution and lies co-planar with the ring and the disc), (ii) the brass outer core (which serves as both the ring contact and main body), (iii) the brass inner core, (the disk contact), (iv) optionally, a stand to assist with assembly, (v) the insulating spacer (which prevents the two brass parts from touching), (vi) the insulating tube (which separates and spaces the brass inner and outer), (vii) the ring and (iix) disk electrodes and (ix) the insulating resin, rapid epoxy, and conductive epoxy. This step can be seen in Figure S1.3a.
2. If using 3D printed parts remove from the supports and remove any defects from printing with a knife or file. This step can be seen in Figure S1.3b.

3. **3.** Dry fit all the parts together to ensure they fit before gluing. Ensure that the brass inner fits flush with the brass outer. If it doesn't, sand the back of the insulating inner until they do. This step can be seen in Figure S1.3c.

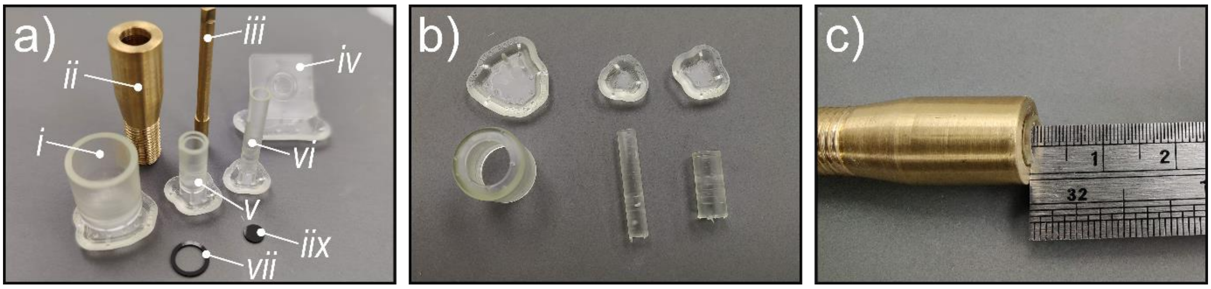

Figure S1.3: Steps 1-3 of RRDE assembly. a) collecting all the parts: i) insulating outer case, ii) brass outer core, iii) brass inner core, iv) RRDE assembly stand, v) insulating spacer, vi) insulating tube, vii) BDD Ring Electrode, iix) BDD Disk Electrode. b) Removing 3D-printed parts from support. c) Checking alignment between inner and outer brass parts with a ruler.

4. Glue all the body parts together with a rapid epoxy, starting with the inner brass core and working outwards. Leave the RRDE face up for 30 minutes to allow the rapid epoxy to set. A spare insulating outer can be used as a stand for the RRDE. This step can be seen in Figure S1.4a.

5. Once set dry fit the ring and disk electrodes into the recesses, which should be sized to match the diameters of the electrodes and ideally be ca. 300 µm deeper than the thickness of the electrodes used. This step can be seen in Figure S1.4b.

6. Mix the conductive epoxy (if using) according to the manufacturer's instructions and apply a small amount to the brass ring and disk contacts. This step can be seen in Figure S1.4c.

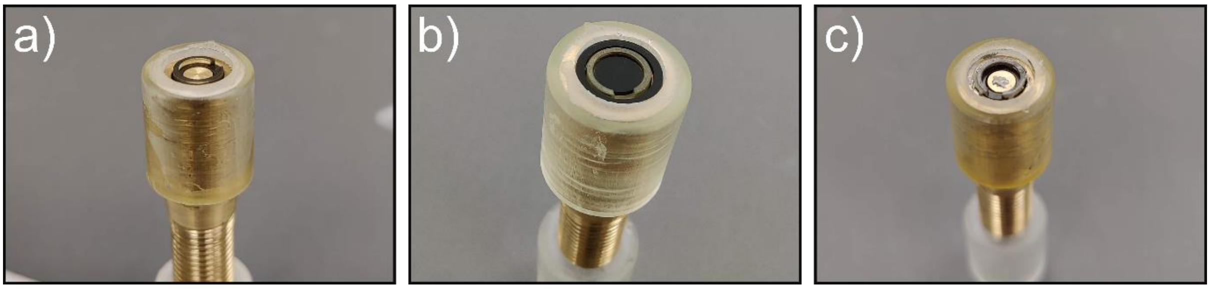

Figure S1.4: Steps 4-6 of RRDE fabrication including, a) attaching the parts together with a rapid epoxy. b) Test fitting the ring and disk electrodes. c) Adding conductive epoxy to the brass contacts.

7. Place the electrodes on top of the conductive epoxy and invert onto the stand. The stand ensures that the electrodes are kept aligned with each other as the epoxy cures. Cure the conductive epoxy according to manufacturer's instructions leaving the RRDE on the stand throughout. This step can be seen in Figure S1.5a.
8. Once the conductive epoxy has set, the RRDE can be removed from the stand and placed face up. The electrode face is then flooded with the insulating epoxy chosen for sealing. It is advantageous to heat the RRDE and resin in a lab oven to an appropriate temperature, to lower the viscosity of the resin and ensure a good seal around the electrodes. Try not to fill the surface with resin past flush as this will make it harder to polish the surface co-planar in later steps. This step can be seen in Figure S1.5b.
9. Cure the insulating resin according to manufacturer's instructions. This step can be seen in Figure S1.5c.

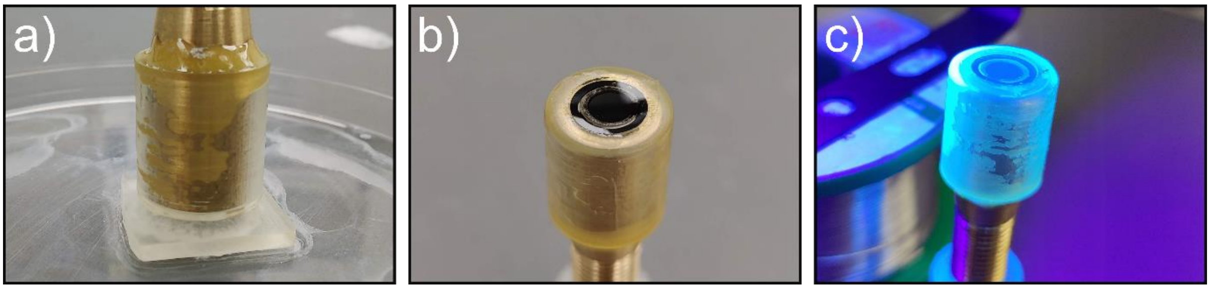

Figure S1.5: Steps 7-9 of RRDE fabrication including, a) curing the conductive epoxy on a stand to ensure the electrodes are co-planar. b) Flooding the electrodes surfaces with insulating resin. c) UV curing the insulating resin.

10. Once the insulating resin is cured it can be polished away to reveal the surface of the electrodes, increasingly fine grades of abrasive paper can be used until the electrode surfaces are completely exposed. Due to the large size of the electrodes, it is easy to polish them flat with some care, pause frequently during polishing and hold the RRDE at right angles to the abrasive paper throughout. This step can be seen in Figure S1.6a and b.

11. After the electrode surfaces are exposed, and typically before each use the surfaces should be alumina polished to ensure the electrodes are clean. This step can be seen in Figure ESI.1.6c.

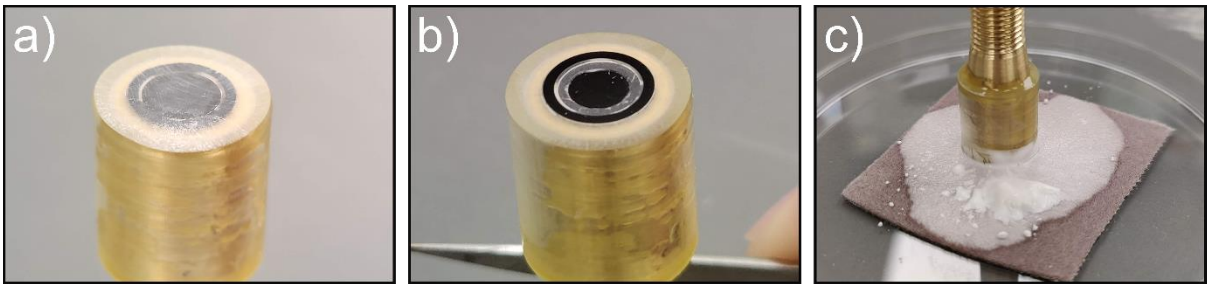

Figure S1.6: Steps 10 and 11 of RRDE fabrication including, a) sanding the insulating resin to expose the electrodes. b) The electrodes are exposed. c) Alumina polish to clean the electrodes.

Rough Electrodes: In the case of a ring or disk electrode with significant surface texture it may be desirable to protect the surface prior to Step 8 where the face is flooded with epoxy, as it may otherwise be impossible to remove all the epoxy from the surface. This could be achieved in two ways; the first method would be to place a piece of tape such as Kapton tape, over the electrode before Step 6. Kapton mask is easy to remove by polishing and should leave no residue. The second method is to flood the surface with PVA Glue before Step 8. The PVA glue is easy to remove by polishing but can also be electrochemically removed by cycling in acid *e.g.* 0.1 M H_2SO_4.

Foil and Rod Materials: While the method of fabrication used was designed for freestanding sheets (wafers) of material or powder compacts (> 100 µm thick) the authors believe it could be easily adapted for other electrode formats. If foil electrode material (< 100 µm thick) was

to be used, we suggest attaching it to both the brass inner and outer between Steps 3 and 4 and then omitting steps 5-7. As metal foils are available at relatively low cost for almost all metals this would be a cost-effective way of making a metal electrode. Using rod material is possible, electrodes could be turned to size on a lathe and the RRDE assembled as normal. If the electrodes are thicker than 360 μm, for which the RRDE parts are designed herein, it would be essential to increase the height of both the insulating inner and outer to be at least 100 μm thicker than the electrode (but ideally 300 μm).

ESI.2 Bill of Materials

Table S1 gives a bill of materials for a single RRDE electrode, excluding the cost of the electrodes themselves.

Table S1: Bill of materials for a single RRDE electrode (bar electrodes).

Part	Estimated Cost Per Use	Notes
Brass Body	\$7 (£5)	Machined in House
Insulating Body (3D Printed)	\$2.50 (£2)	Printed on a Form 3
Insulating Body (PEEK)	\$7 (£5)	Machined in House
Silver Epoxy	\$2.50 (£2)	Chemtronics CW2400 Conductive Adhesive
Epoxy Resin	< \$4 (£3)	If the body is SLA printed that UV resin should be used for sealing as this will ensure adhesion. If PEEK is used a resin with good adherence to PEEK should be used.
**Total:**	< \$25 (£20) Per RRDE	Not including electrodes or assembly time.

ESI.3 CVs of RRDE Electrodes in 1 mM $FcTMA^+$ and 0.1 M KNO_3

Figure S2 shows CVs in the redox mediator $FcTMA^+$ for the ring and disk electrodes of both RRDEs.

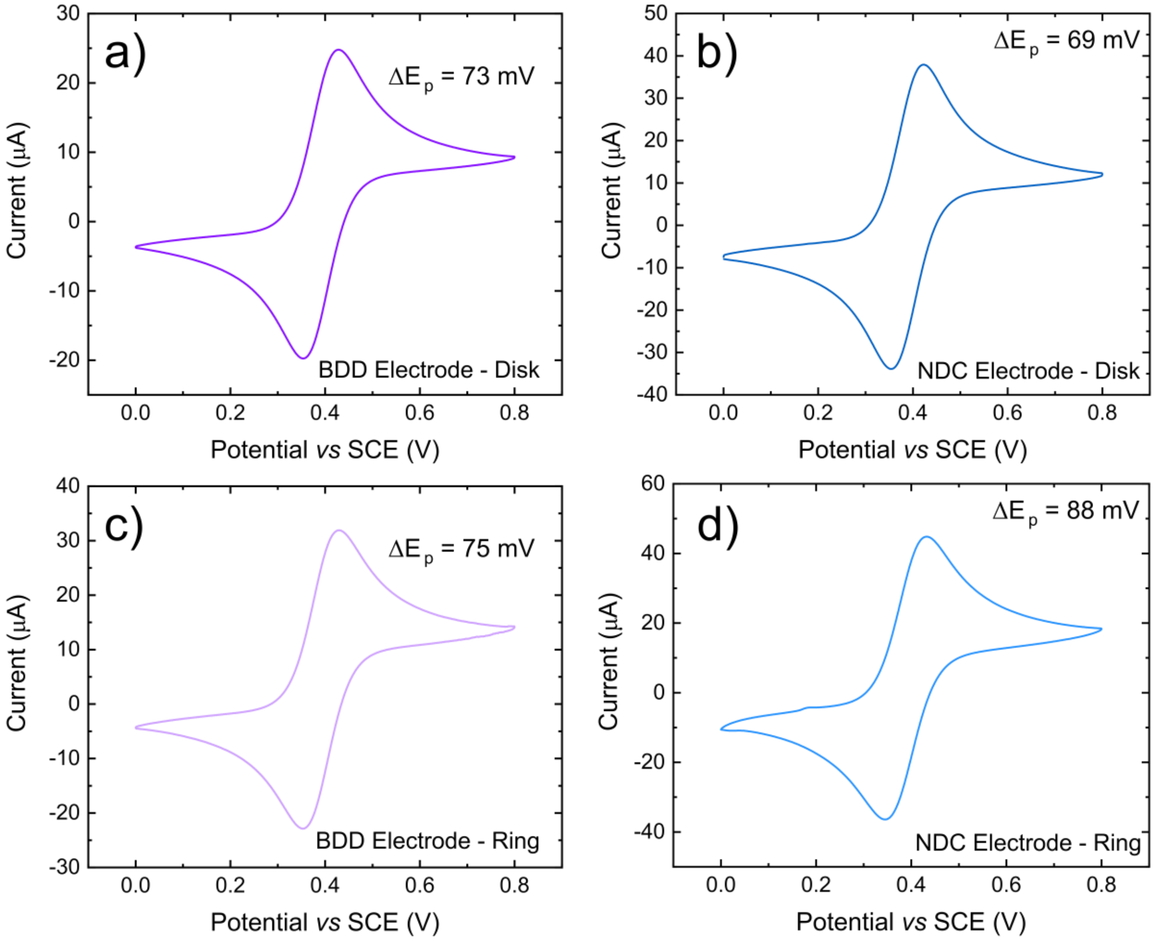

Figure S2: CVs at 100 mV s^{-1} for ring and disk electrodes in 1 mM $FcTMA^+$ in 0.1 M KNO_3. a) BDD disk electrode. b) NDC-BDD disk electrode. c) BDD ring electrode (for BDD disk RRDE). d) BDD ring electrode (for NDC-BDD disk RRDE). Presented data is the third scan of three.

ESI.4 Uncompensated Resistance Measurements

Uncompensated resistance, R_u, measurements were made by collecting the i-t data from a series of potential pulses in the non-faradaic region (± 0.1 V) of all electrodes in 0.1 M KNO_3 (*n*=5). To collect sufficient data points over the timescale of the i-t decay curve a potentiostat fitted with a fast scan module was used (PGSTAT128N with ADC10M.S, Metrohm Autolab, Swizerland). The i-t response was fitted to Equation S1,[1] where ΔE represents the height of the potential pulse and C_{dl} is the electrochemical double layer capacitance, enabling values of both C_{dl} and R_u to be obtained.

Equation S1:

$$i(t) = \frac{\Delta E}{R_u} e^{-t/R_u C_{dl}}$$

The R_u values for the five pulses (Figures S3 and 4) per electrode were then averaged to obtain the value for R_u presented in Table S2.

Table S2 Uncompensated Resistance Values

**Electrode**	**R_u (Ω)** n=5	**Ru Error (Ω)**
BDD Disk	100	± 1
BDD Ring	61	± 1
NDC-BDD Disk	114	± 5
BDD Ring (NDC-BDD Disk)	59	± 1

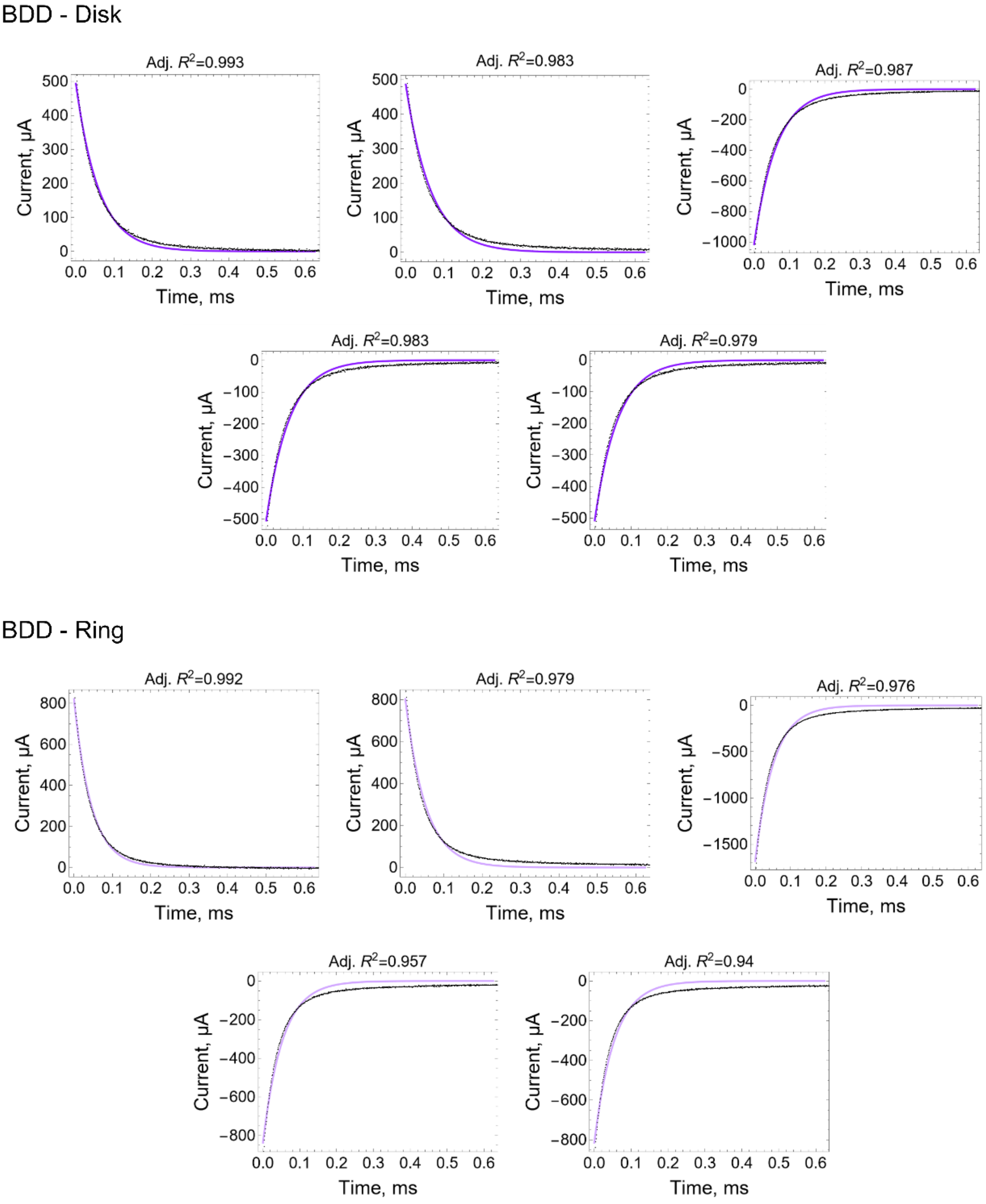

Figure S3: i-t experimental non-faradaic decay data (black points) with fits (purple line, equation S1) from which R_u values were extracted for the BDD RRDE electrode.

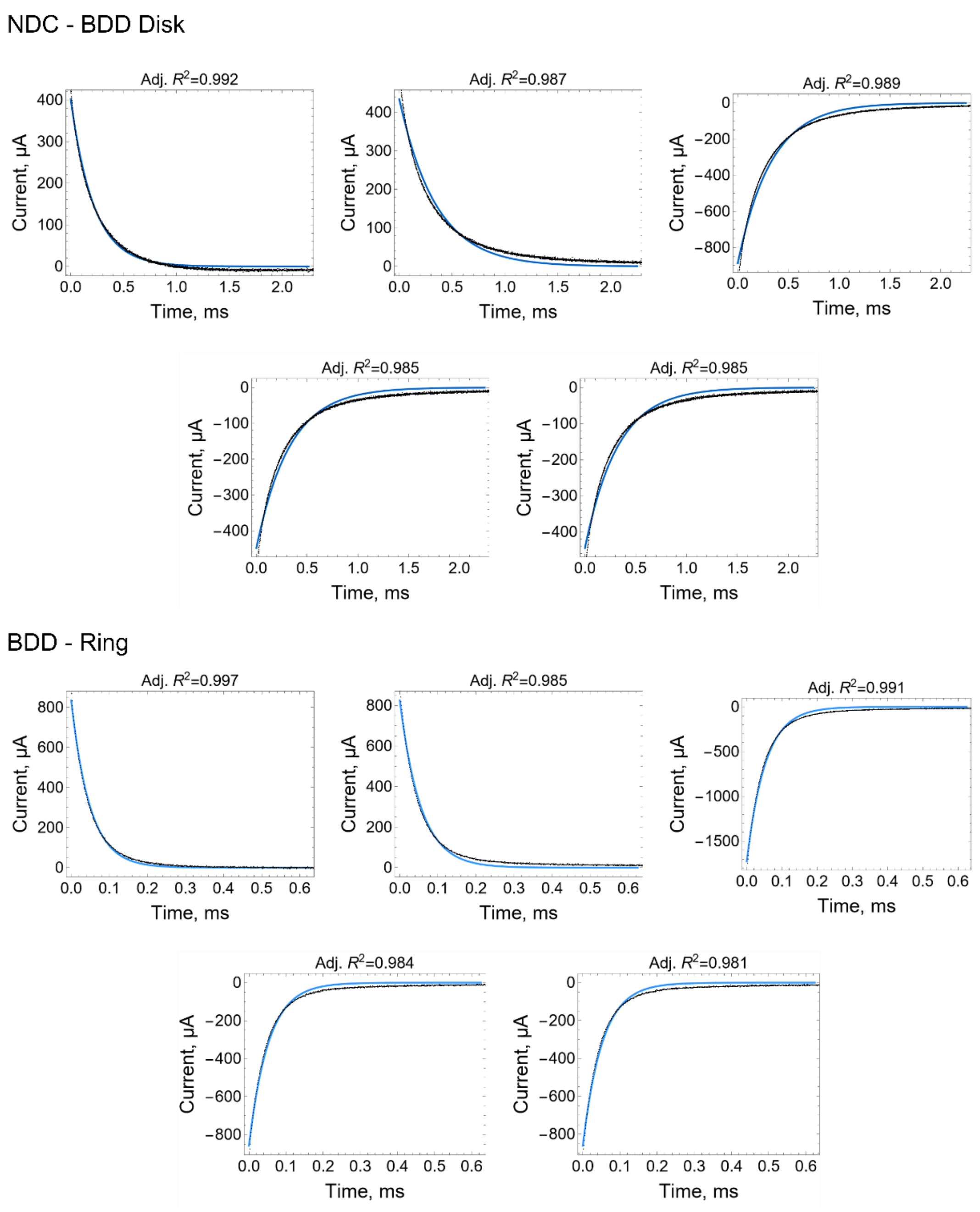

Figure S4: i-t experimental non-faradaic decay data (black points) with fits (blue line, equation S1) from which R_u values were extracted for the NDC-BDD (disk), BDD ring RRDE electrode.

ESI.5 White Light Interferometry (WLI) of Disk Electrode Surfaces

WLI images of both the BDD (Figure S5a) and NDC-BDD (Figure S5b) disk electrodes were collected using a Bruker ContourGT (Bruker Nano Inc, USA) using a 5× objective. Data was processed in Gwydion 2.55. The plane was levelled and then a 4th order polynomial background subtracted before roughness measurements were taken. Roughness values are taken as RMS from the entire image. Figure ESI.4b shows the raster of the laser used to create the sp^2 bonded carbon layer on the surface.

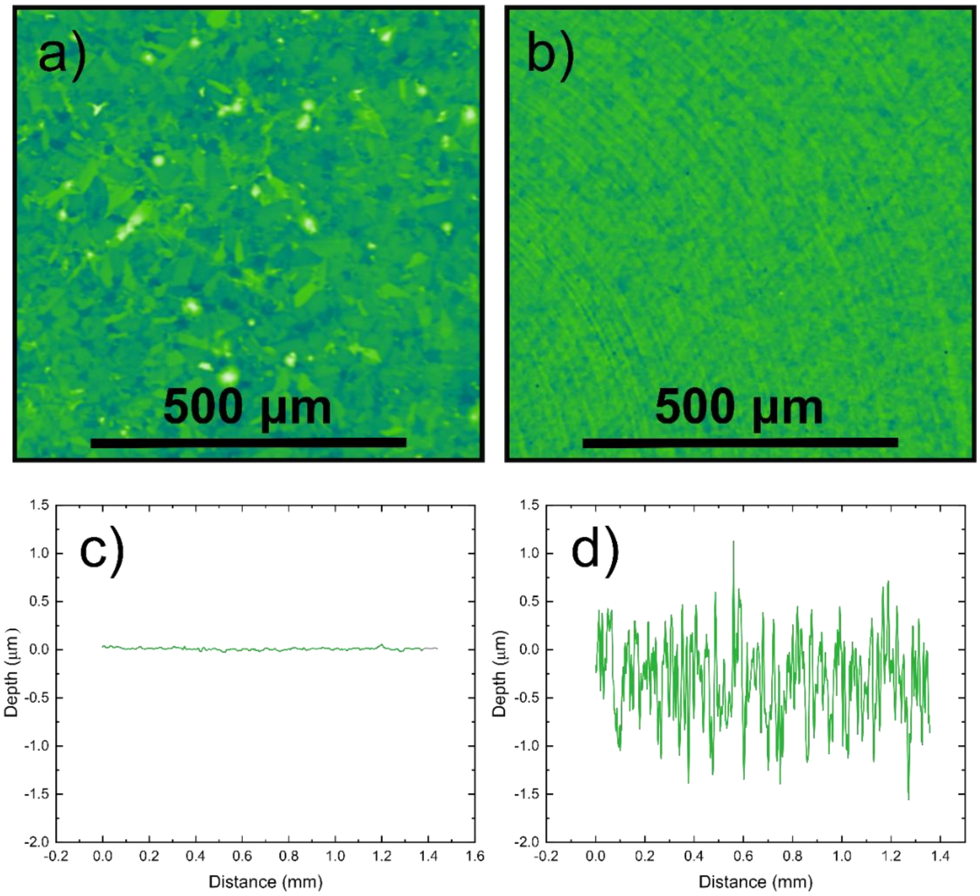

Figure S5. WLI images of a) the BDD disk electrode, giving an RMS of 10 nm. b) The NDC-BDD disk electrode, giving an RMS of 460 nm. The circular tracks in this image are the raster of the laser micromachining system. c) Line profile diagonally across a showing the surface height variation. d) Line profile diagonally across b showing the surface height variation.

ESI.6 Quinone Surface Coverage (QSC) Measurements

QSC measurements were performed to quantify the difference in NDC content between the BDD and NDC-BDD disk electrodes following the protocol described in reference.[2] It has been previously shown that QSC measurements show a high degree of correlation with surface NDC both as-grown and from incorporation by laser micromachining.[2] The results of the QSC measurements can be seen in Figure S6. These measurements were collected using digital staircase voltammetry.

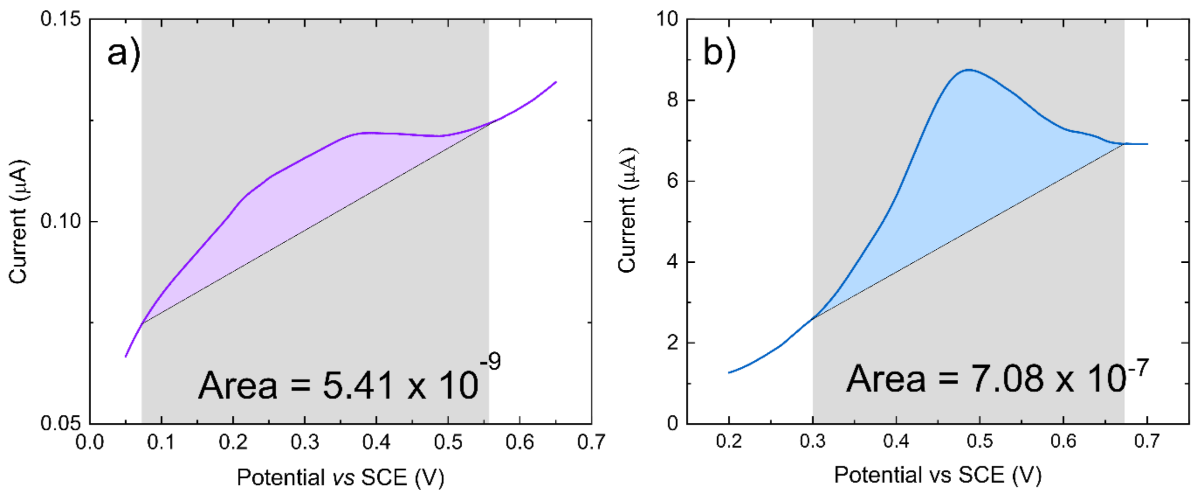

Figure S6: QSC Measurements of a) BDD b) NDC-BDD disk electrodes. Measurements conducted in pH 2 Carmody Buffer at 0.1 V s^{-1}. Note that a different integration window had to be chosen for each electrode due to the difference in peak shape and position.

The area under the quinone oxidation peak was converted to a quinone surface coverage using Equation S2. i_A is the integrated area under the peak, n is the number of electrons transferred, A is the area of the electrode measured from WLI, and v is the scan rate.

Equation S2:

$$\Gamma = \frac{i_A}{nAFv}$$

This gave a QSC of 2.0×10^{-12} mol cm^{-2} for the BDD disk electrode and a QSC of 2.6×10^{-10} mol cm^{-2} for the NDC-BDD disk electrode.

ESI.7 Generation Collection Response of the NDC-BDD disk and BDD ring electrode (RRDE)

The collection efficiencies of the RRDE for the redox couple $FcTMA^{+}$ / $FcTMA^{2+}$ were measured for comparison with the theoretical efficiency. The potential of the NDC-BDD disk electrode was scanned from a value where no electron transfer occurred (0.00 V vs SCE) to one where the oxidation of $FcTMA^{+}$ to $FcTMA^{2+}$ was mass transport limited. The potential of the BDD ring electrode was held at a value where reduction of $FcTMA^{2+}$ was also mass transport limited (0.05 V vs SCE). The experiments were performed as a function of rotation rate from 1000 RPM to 2000 RPM in 500 RPM steps, which can be seen in Figure S7.

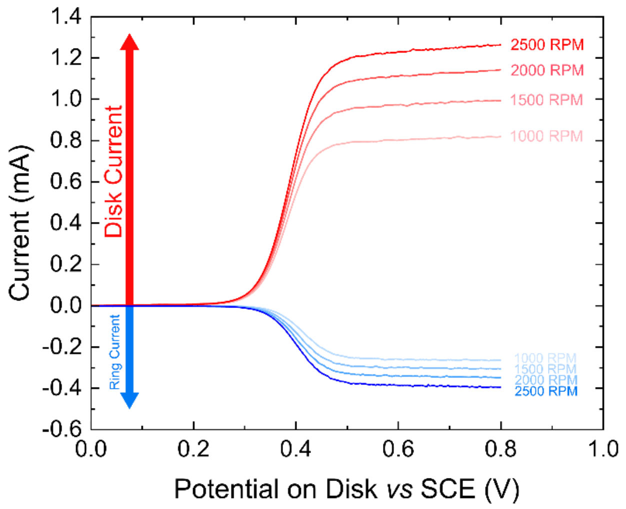

Figure S7: LSVs on the disk recorded at 100 mV s^{-1} in 0.1 M KNO_3 for the NDC-BDD disk in the RRDE. Ring held at 0.05 V *vs* SCE.

ESI.8 Ring and Disk Currents at 0.60 V vs SCE

The theoretical collection efficiency for the RRDE design employed were calculated using the spreadsheet provided by Pine Research (https://pineresearch.com/shop/wp-content/uploads/sites/2/2017/02/RRDE-Collection-Efficiency-Calculator-Worksheet-REV003.xlsx). Calculations are based on the those detailed in section 9.4 of Bard and Faulkner.[3] The disk electrode has a diameter of 5.00 mm, the ring has an inner and outer diameter of 7.00 and 9.00 mm respectively. This gives rise to a theoretical collection efficiency of 35%. The empirical collection efficiencies for the BDD RRDE and NDC-BDD RRDE can be seen in tables S3 and S4 respectively.

Table S3: Ring and Disk Currents and Collection Efficiencies for the BDD RRDE.

Speed (RPM)	Disk Current (μA)	Ring Current (μA)	Collection Efficiency (%)
1000	0.544	-0.171	31
1500	0.655	-0.201	31
2000	0.733	-0.239	33
2500	0.867	-0.265	32
			**Average = 32**

Table S4 Ring and Disk Currents and Collection Efficiencies for the NDC-BDD disk and BDD ring RRDE

Speed (RPM)	Disk Current (μA)	Ring Current (μA)	Collection Efficiency (%)
1000	0.812	-0.262	32
1500	0.987	-0.305	31
2000	1.123	-0.345	31
2500	1.249	-0.391	31
			**Average = 31**

ESI.9 Levich Analysis

Levich analysis was used to assess the performance of the RDEs in the RRDE. A limiting current versus rotation rate$^{1/2}$ plot should be linear. From this line determination of the diffusion coefficient of the redox active analyte ($FcTMA^+$) is possible. Measurement of the diffusion coefficient of $FcTMA^+$ in the same supporting electrolyte (0.1 M KNO_3) using a 25 µm platinum ultramicroelectrode (UME) gave a value of 5.1×10^{-6} cm^2 s^{-1}.

The disk electrode currents on both RRDEs were plotted against rotation rate$^{1/2}$ to extract diffusion coefficients for $FcTMA^+$. A disk electrode with ideal hydrodynamics should obey the Levich equation, Equation S3,[3] where I_L is the limiting current, *n* is the number of electrons transferred, F is Faraday's constant, A is the electrode area, D is the diffusion coefficient, ω is the rotation rate, υ is the kinematic viscosity of the solution, and C is the concentration of the analyte. The Levich plot for the BDD disk electrode can be seen in Figure S8.

Equation S3:

$$I_L = (0.620)nFAD^{\frac{2}{3}}\omega^{\frac{1}{2}}\upsilon^{\frac{-1}{6}}C$$

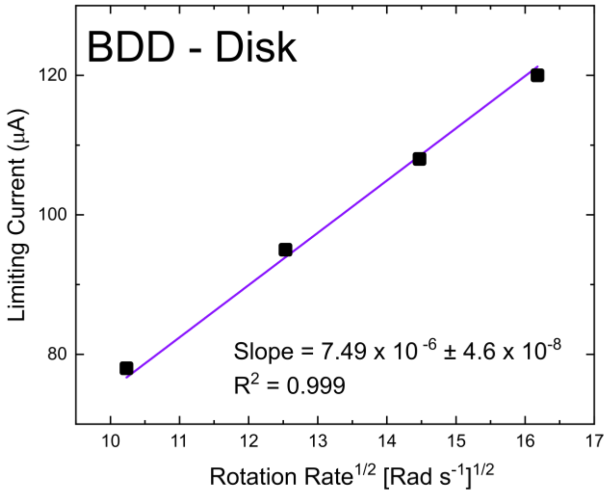

Figure S8: Levich analysis of the BDD disk electrode with 1 mM $FcTMA^+$ in 0.1 M KNO_3 at rotation rates from 1,000 to 2,500 RPM. Note the Y intercept is fixed at 0.

Levich analysis of the BDD disk gives an R^2 of 0.999, demonstrating that this electrode performs in good agreement with theory. Extracting a D value from the gradient gives 5.1×10^{-6} cm^2 s^{-1}, which is in agreement with that measured using the UME in the same solution.

The Levich plot for the NDC-BDD disk can be seen in Figure S9.

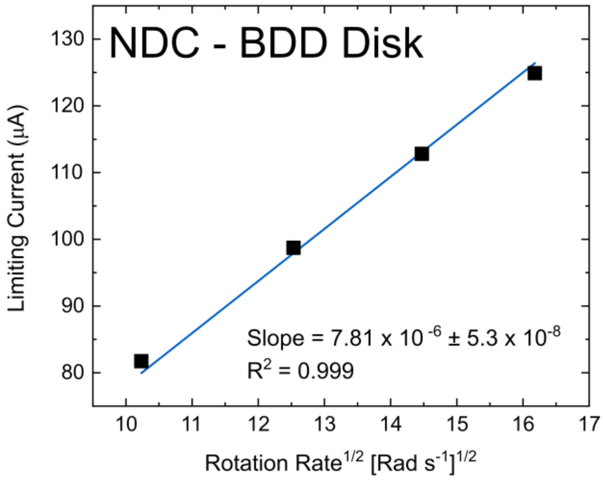

Figure S9: Levich analysis of the NDC-BDD disk electrode with 1 mM $FcTMA^+$ in 0.1 M KNO_3 at rotation rates from 1,000 to 2,500 RPM. Note that the Y intercept is fixed at 0.

Levich analysis of the NDC-BDD disk demonstrates a similar adherence to theory with an R^2 of 0.999. Extracting a $FcTMA^+$ diffusion coefficient from this data gives a value of 4.9×10^{-6} cm^2 s^{-1}.

ESI.10 SEM of Pt-NP Modified Ring Electrode

Field emission scanning electron microscopy (FE-SEM) images of the Pt-NP modified BDD ring electrode was taken using the SE2 secondary electron detector of a Zeiss Supra 55VP FE-SEM (Zeiss, Germany) operating at 10 kV (Figure S10). The FE-SEM image revealed a high-density of Pt NPs, 10's of nm in size, with preferential deposition observed on the more highly doped grains of the BDD.

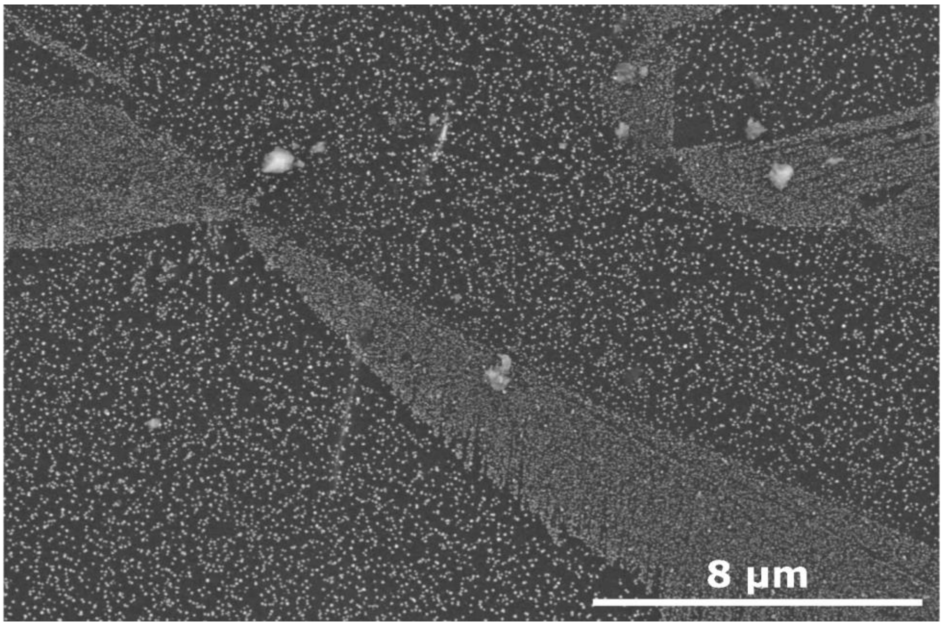

Figure S10: FE-SEM image of the Pt-NP modified BDD ring electrode.

ESI.11 BDD Disk Electrode Ring Calibrations for H_2O_2

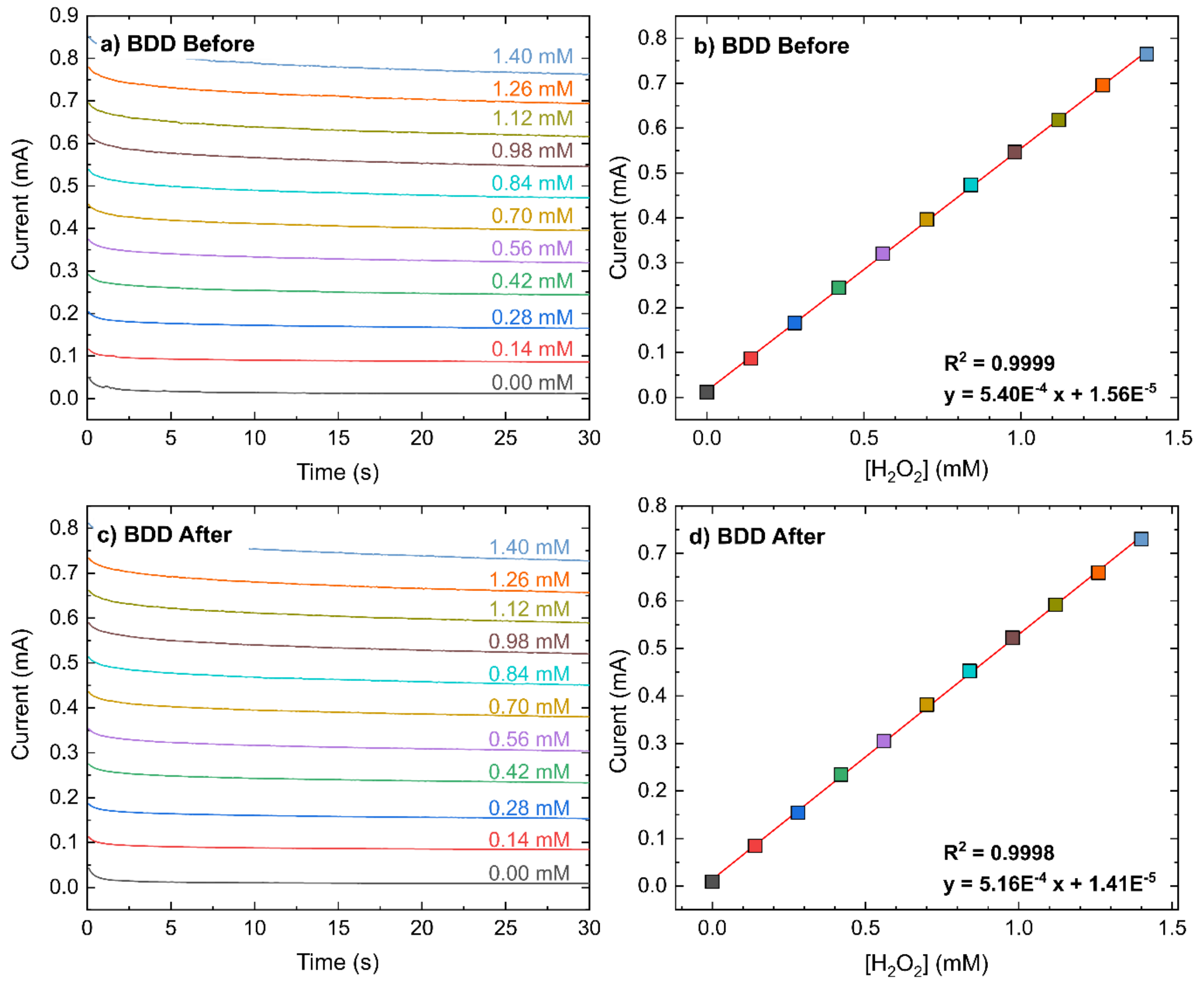

Figure S11: Before and after calibrations for the Pt NP deposited ring on the RRDE with BDD disk electrode. Ring held at 1.00 V *vs* SCE and H_2O_2 added via standard addition. a) Current-time responses for the ring before generation-collection experiments. b) Calibration plot of the average current over the last 5 s *vs* H_2O_2 concentration before generation-collection experiments. c) Current-time responses for the ring after generation-collection experiments. d) Calibration plot of the average current over the last 5 s *vs* H_2O_2 concentration after generation-collection experiments. The ring was calibrated before and after generation-collection experiments to ensure that the before calibration was still valid.

ESI.12 NDC BDD Disk Electrode Ring Calibrations for H_2O_2

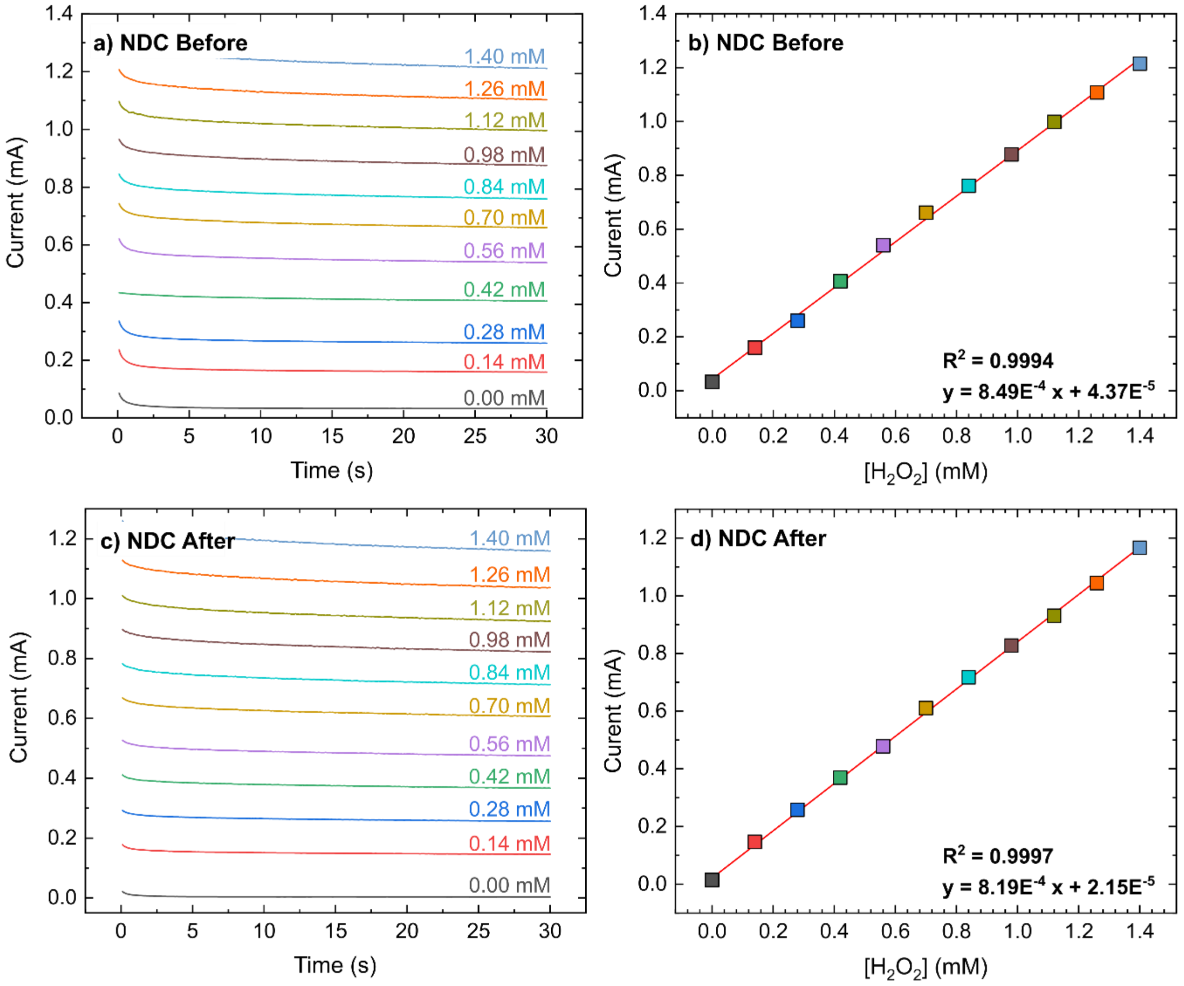

Figure S12: Before and after calibrations for the Pt NP deposited ring on the RRDE with NDC-BDD disk electrode. Ring held at 1.00 V *vs* SCE and H_2O_2 added via standard addition. a) Current-time responses for the ring before generation-collection experiments. b) Calibration plot of the average current over the last 5 s *vs* H_2O_2 concentration before generation-collection experiments. c) Current-time responses for the ring after generation-collection experiments. d) Calibration plot of the average current over the last 5 s *vs* H_2O_2 concentration after generation-collection experiments. The ring was calibrated before and after generation-collection experiments to ensure that the before calibration was still valid.

ESI.13 Faradic Efficiency

Faradaic efficiency of H_2O_2 generation on both disk electrodes was calculated at -0.6, -0.7, and -0.8 V *vs* SCE, according to Equation S3.[3] Where i_{ring} is the current measured on the ring, i_{Disk} is the current measured on the disk, and $N_{Empirical}$ is the empirical (measured) collection efficiency for the electrode used.

Equation S3: $$\%FE = \frac{i_{Ring}}{i_{Disk}\, N_{Empirical}}$$

Table S5: Faradaic efficiencies for the generation of hydrogen peroxide

Disk Electrode	% CE	Potential (V) *vs* SCE	Disk Current (mA)	Ring Current (mA)	% FE
BDD	32	- 0.6	0.0646	0.0054	**26%**
		-0.7	0.1814	0.0078	13%
		-0.8	0.3980	0.0011	1%
NDC-BDD	31	- 0.6	0.2154	0.0427	**64%**
		-0.7	0.4511	0.0697	50%
		-0.8	0.9241	0.0868	30%

References

(1) Cobb, S. J.; Macpherson, J. V. Enhancing Square Wave Voltammetry Measurements via Electrochemical Analysis of the Non-Faradaic Potential Window. *Anal. Chem.* **2019**, *91* (12), 7935–7942. https://doi.org/10.1021/acs.analchem.9b01857.

(2) Ayres, Z. J.; Cobb, S. J.; Newton, M. E.; Macpherson, J. V. Quinone Electrochemistry for the Comparative Assessment of Sp2 Surface Content of Boron Doped Diamond Electrodes. *Electrochem. commun.* **2016**, *72*, 59–63. https://doi.org/10.1016/j.elecom.2016.08.024.

(3) Bard, A. J.; Faulkner, L. R. *Electrochemical Methods - Fundamentals and Applications*, 2nd ed.; Wiley, 2001; Vol. 2.
